# Outcomes analysis of breast reduction in Brazilian women using the BREAST-Q^®^ questionnaire: a cross-sectional controlled study

**DOI:** 10.6061/clinics/2018/e313

**Published:** 2018-06-12

**Authors:** Adriana Corbolan Andrade, Daniela Francescato Veiga, Isabella de Carvalho Aguiar, Yara Juliano, Miguel Sabino, Lydia Masako Ferreira

**Affiliations:** I Programa de Pós-graduação em Cirurgia Translacional, Universidade Federal de Sao Paulo, Sao Paulo, SP, BR; II Departamento de Bioestatistica, Universidade do Vale do Sapucai, Pouso Alegre, MG, BR

**Keywords:** Breast, Mammoplasty, Patient’s Satisfaction, Evaluation of Results, Quality of Life

## Abstract

**OBJECTIVE:**

The aim of this study was to analyse patient-reported outcomes of reduction mammoplasty among Brazilian women.

**METHODS:**

A total of 100 women were enrolled in this cross-sectional controlled study, 50 with breast hypertrophy (Hypertrophy Group) and 50 who had undergone breast reduction at least six and up to 12 months before (Mammoplasty Group). The Brazilian version of the BREAST-Q^®^ was applied to assess patient-reported outcomes. The module reduction/mastopexy was used, and the preoperative and postoperative versions were applied to the hypertrophy and mammoplasty groups, respectively.

**RESULTS:**

The mammoplasty group presented higher scores for the subscales satisfaction with breasts, psychosocial well-being, sexual well-being and physical well-being (p=0.0001 for all of these subscales).

**CONCLUSION:**

These results suggest that patients submitted to reduction mammoplasty are satisfied with the outcomes and present better quality of life scores compared with women with breast hypertrophy.

## INTRODUCTION

The concept of breast hypertrophy goes beyond the simple characterization of breast size. Breast hypertrophy can be defined as an increase of the mammary gland beyond the physiological limits, with the exception of increases caused by injuries, haemorrhages, inflammation and pregnancy (1,2).

Patients seeking breast reduction do so with the hope of obtaining a better quality of life, with less social and sexual embarrassment and greater ease in performing physical activities and in finding suitable clothes (3). Women with breast hypertrophy may suffer from low self-esteem and seek surgery to alleviate physical and emotional discomfort (3). Reduction mammoplasty is very effective in improving functional, aesthetic and psychological problems, and several studies have demonstrated its effectiveness in improving quality of life (3-7).

According to the International Society of Aesthetic Plastic Surgery (ISAPS), in 2016, Brazil ranked second in the *ranking* of plastic surgery procedures in the world, with 1,450,020 surgeries performed. Specifically, breast reduction was the eighth most performed surgery by plastic surgeons worldwide, with 465,665 mammoplasties total. Of these, 78,815 were performed in Brazil. Therefore, it is important to evaluate the satisfaction results of Brazilian women undergoing reduction mammoplasty (8). However, the benefits of this procedure have rarely been quantified in an objective and standardized manner (3,5,7).

BREAST-Q^®^ was developed for the evaluation of results in breast surgery, aiming to identify the best procedures for a given patient and the procedures that provide the greatest satisfaction (9,10).The objective of this controlled cross-sectional study was to evaluate the effects of reduction mammoplasty in Brazilian women with breast hypertrophy from the perspective of the patients. To the best of our knowledge, this study is unique because it used BREAST-Q^®^ to compare the satisfaction and quality of life of women submitted to reduction mammoplasty with women with breast hypertrophy who did not seek the procedure for any reason.

## METHODS

This cross-sectional study was approved by the Ethics on Research Committee of the Universidade Federal de São Paulo under protocol 165302/12, and all participants signed a free and informed consent form. The sample size was estimated based on studies of other outcomes in reduction mammoplasty and was sufficient to obtain significant results (11-13).

Fifty women with breast hypertrophy (Hypertrophy Group, HG) defined by the criteria of Sacchini et al. and Franco & Rebello and 50 women previously submitted to reduction mammoplasty (Mamamoplasty Group, MG) at least six months and at most one year prior to the study’s initiation were selected from the plastic surgery outpatient clinics of a university hospital (Hospital São Paulo), between January 2014 and January 2015 (16,17).

Inclusion criteria for both groups were age between 18 and 45 years and body mass index (BMI) between 19 and 29.9 kg/m^2^, without restrictions regarding ethnicity, schooling or social class. In the HG, patients with previous breast surgeries were not included, and in the MG, patients who underwent mammoplasty less than six months or more than one year prior to the study’s initiation were not included. The exclusion criteria for both groups were illiteracy or inability to read and understand the applied questionnaire, pregnancy or childbirth less than one year ago and currently being investigated for or diagnosis of breast disorders.

Women who met the eligibility criteria were informed about the study and were invited to participate. After signing the informed consent, sociodemographic and clinical data were collected, and the Brazilian version of BREAST-Q^®^ was self-administered.

BREAST-Q^®^ was developed in 2009 to assess the impact and effectiveness of breast surgeries from the patient’s perspective. It was translated and validated for use in Brazil in 2013 (18). The questionnaire consists of five modules – augmentation mammoplasty, reduction mammoplasty, mastectomy, breast reconstruction and conservative treatment – and there is one version for the pre- and one for the postoperative period. Each module is composed of independent subscales: Physical well-being, Psychosocial well-being, Sexual well-being, Satisfaction with breasts, Satisfaction with nipples, Satisfaction with the overall outcome and Satisfaction with the care process. The answers are transformed using Q-Score^®^ scoring software, with total scores ranging from zero to 100. The higher the score is, the greater the satisfaction or the better the quality of life (19).

### Statistical analysis

For the statistical analysis, the *software* BioEstat 5.0 (Instituto de Desenvolvimento Sustentável Mamirauá, Belém, PA, Brazil) was used. The non-parametric Mann-Whitney test was used to compare the two independent groups for the numerical variables studied. We also performed a simple linear regression analysis to study relationships between BMI (independent variable) and the subscale “Satisfaction with breasts” (dependent variable). The level of significance was set at 0.05 or 5%.

## RESULTS

**Figure 1:**
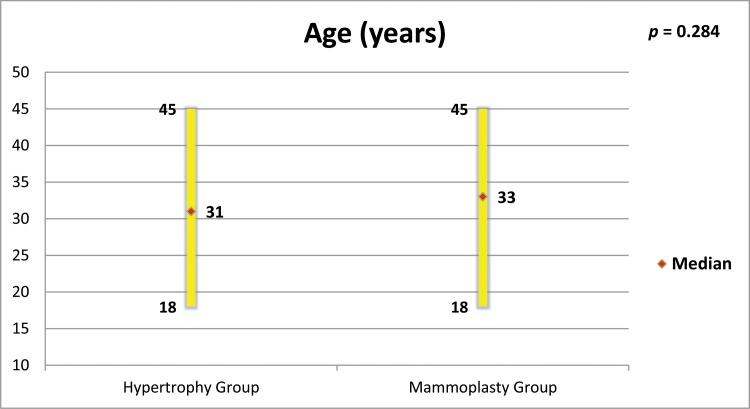
Age of women in both groups and comparison by the Mann-Whitney test.

**Figure 2:**
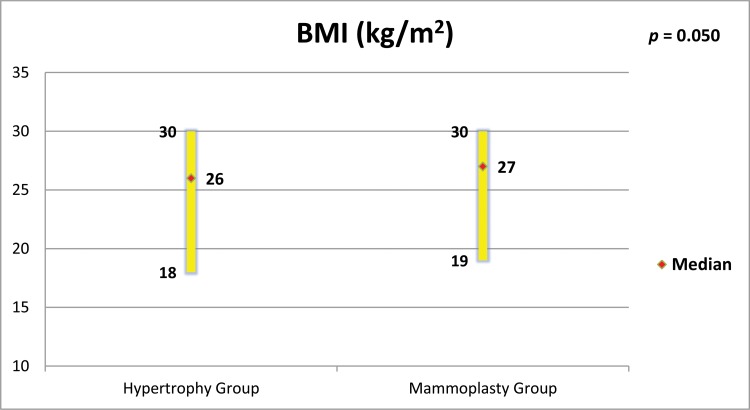
Body Mass Index (BMI) of women in both groups and comparison by the Mann-Whitney test.

**Figure 3:**
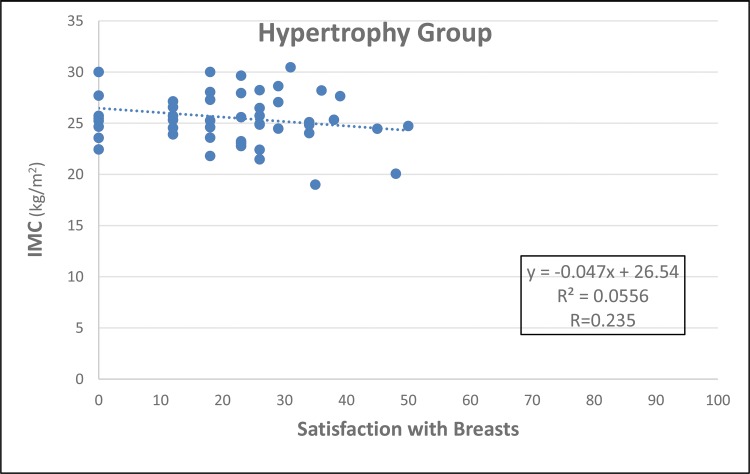
Simple linear regression for BMI (independent variable) and “Satisfaction with breasts” (dependent variable) in the HG.

**Figure 4:**
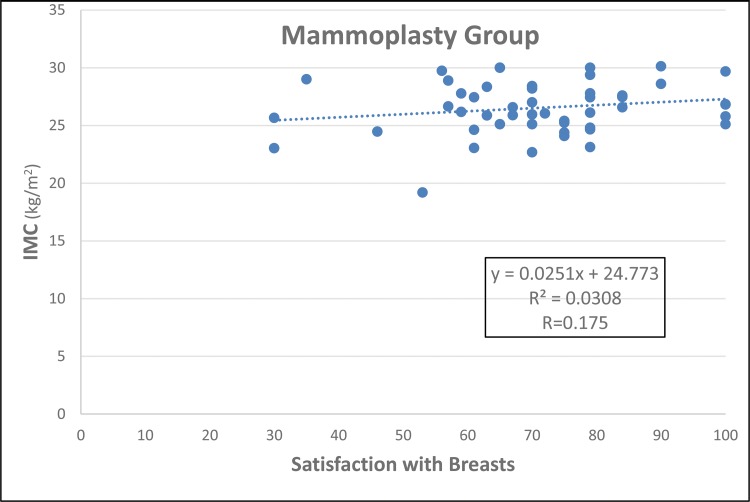
Simple linear regression for BMI (independent variable) and “Satisfaction with breasts” (dependent variable) in the MG.

The groups were age-matched (*p*=0.284, Figure 1). MG patients had a higher BMI (*p*=0.050, Figure 2), but there was no important relationship between BMI and “Satisfaction with breasts” (Figures 3 and 4). In the MG, the total weight of resected breast tissue ranged from 280 to 3,750g (median: 830g; mean±standard deviation: 1107±834g).

**Figure 5:**
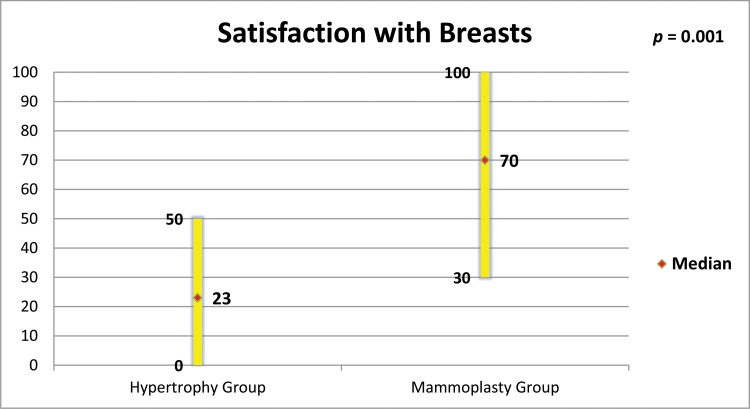
Scores of BREAST-Q^®^ subscale “Satisfaction with breasts” in both groups and comparison by the Mann-Whitney test.

**Figure 6:**
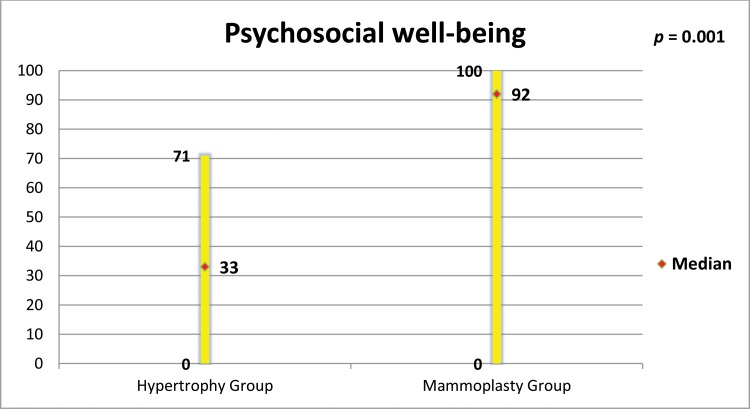
Scores of BREAST-Q^®^ subscale “Psychosocial well-being” in both groups and comparison by the Mann-Whitney test.

**Figure 7:**
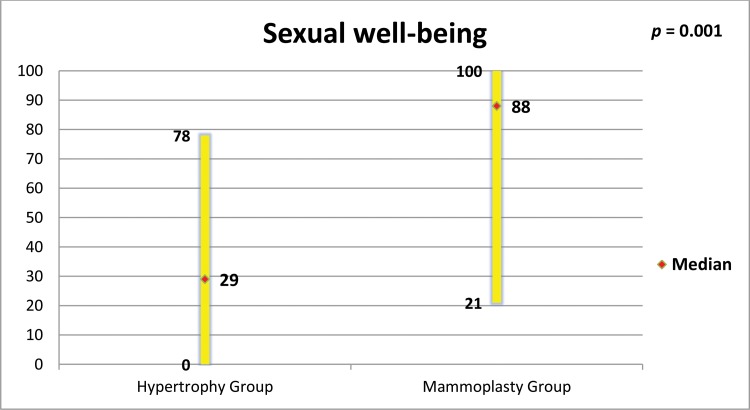
Scores of BREAST-Q^®^ subscale “Sexual well-being” in both groups and comparison by the Mann-Whitney test.

**Figure 8:**
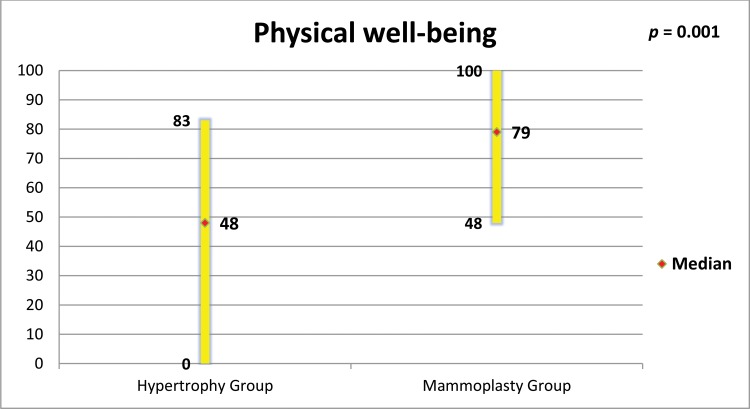
Scores of BREAST-Q^®^ subscale “Physical well-being” in both groups and comparison by the Mann-Whitney test.

**Figure 9:**
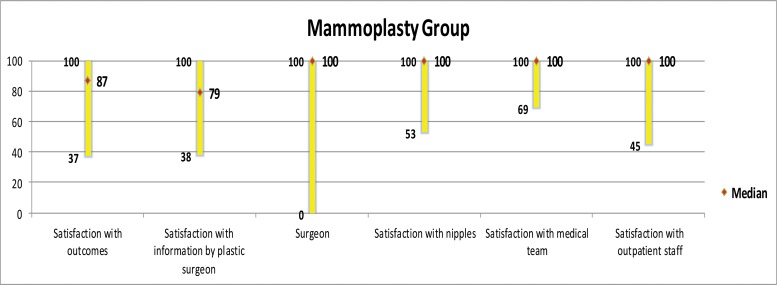
Scores of BREAST-Q^®^ subscales applied to the MG only.

Figures 5 to 8 present the comparisons between the HG and MG with regard to the scores obtained for the four subscales of BREAST-Q^®^ applied to both groups (Satisfaction with breasts, Physical well-being, Psychosocial well-being and Sexual well-being). The MG presented better outcomes in all of these subscales. Figure 9 presents the range and median scores obtained for the subscales of BREAST-Q^®^ applied to the MG only.

## DISCUSSION

The results in plastic surgery are evaluated in terms of not only morbidity and mortality, but mainly patient satisfaction, and the surgeon’s perception of the outcome is often different from the patient’s perception (20-22). The importance of understanding the patient’s perception about the surgical outcome and the impact that plastic surgery can have on the patient’s quality of life is being increasingly recognized. This recognition has led to the development of instruments called PROs - Patient Reported Outcomes, which can provide important information for health policy decision-making (23).

The present study proposed to use a widely applied PRO instrument, the Breast-Q, to evaluate the results of reduction mammoplasty. Women between 18 and 45 years of age were selected for the study. The cut-off of 18 years was chosen to include only adult patients who could spontaneously participate in the study and with their own consent. The 45-year age cut-off was chosen because it was not intended to include perimenopausal or menopausal patients because these patients present hormonal changes that are characteristic of this period and that may lead to alterations in sexual function, thus interfering in the outcomes of study (24).

The BMIs of patients with breast hypertrophy are usually higher than those of patients without hypertrophy, and the patient’s BMI tends to be higher the greater the hypertrophy (25,26). It was decided not to include women with BMIs above 30 kg/m^2^ in any of the groups in this study, and this eligibility criterion was the main excluding factor in the study, which made selection difficult. However, the criterion was maintained to minimize bias because patients with larger BMIs present changes in their centre of gravity and alterations in bone joints that can cause discomfort and pain, which could be confounding factors in the results (2). In the current study, women in the MG had higher BMIs than those in the HG. However, the linear regression showed no important relationships between BMI and satisfaction with breasts in both groups.

The choice of the sixth postoperative month as the minimum time to evaluate the patients in the MG was because the eventual complications and discomfort characteristic of the postoperative period have already been overcome after this period. After six months, the euphoria and the overestimation of the outcomes that usually occur soon after surgery have stabilized (3,4,14,15). The cut-off of one year postoperatively was established to avoid the possibility that, in a very late evaluation, the patient no longer remembered details of the treatment they received.

The MG presented large variations in the total weight of resected breast tissue (280 to 3,750g). Studies have shown that patients with breast hypertrophy usually show improvement of symptoms, regardless of the volume of resected breast tissue (15,28-30). GONZALES *et al*. used the BREAST-Q^®^ to evaluate the results of 600 patients submitted to reduction mammoplasty. They observed, as in the present study, better outcomes in all BREAST-Q^®^ subscales, and more than 95% of the surveyed patients were satisfied with the surgery and would have it performed again. In addition, they observed that BMI and breast size had no influence on outcome (31).

The high scores of the physical well-being scale observed in this study support what has been observed by other authors, who verified that reduction mammoplasty was able to promote improvements in functional capacity, back pain, work capacity and productivity among women with breast hypertrophy (2,27,32).

In the present study, high scores were also obtained for the “satisfaction with nipple-areolar complex” scale. Garcia et al. reported that reduction mammoplasty reduced the sensitivity of the nipple-areolar complex, but did not influence sexual function (33). Beraldo et al. observed a positive impact of reduction mammoplasty on the sexual function of women with breast hypertrophy, a result also corroborated by the high scores in the sexual well-being scale found in the present study (24). A significant portion of the Brazilian population depends on the Brazilian public health system (Sistema Único de Saúde - SUS), which is often the only option for women with breast hypertrophy. Araújo et al. studied the cost-utility relationship of reduction mammoplasty performed by the SUS and found that there was a positive relationship, justifying the need to mobilize resources for this type of procedure (34).

This study has some limitations. The main limitation is the cross-sectional design. A prospective study, with pre and postoperative assessment, would be able to detect the real impact of breast reduction on patientś quality of life. Another limitation is the lack of a group of women with normal-sized breasts for comparison to women with breast hypertrophy and breast reduction. However, no other study was found in the literature using BREAST-Q^®^ to compare the satisfaction and quality of life of women submitted to reduction mammoplasty with women with breast hypertrophy who did not undergo the procedure for any reason, making this study unique.

Our results suggest that patients submitted to reduction mammoplasty are satisfied with the outcomes and present better quality of life scores when compared with women with breast hypertrophy. However, prospective studies are needed to confirm these findings.

## AUTHOR CONTRIBUTIONS

Andrade AC and Aguiar IC were responsible for data collection and preparation of the manuscript. Veiga DF and Juliano Y were responsible for data analysis and preparation of the manuscript. Veiga DF and Sabino-Neto M edited the manuscript. Veiga DF and Ferreira LM supervised the study and were responsible for manuscript editing. All of the authors read and approved the final version of the manuscript.
